# Editorial: Tumor accommodation: the importance of the niche in neurological tumors

**DOI:** 10.3389/fonc.2024.1383594

**Published:** 2024-03-05

**Authors:** Roberta Azzarelli, Laurent R. Gauthier, Jose R. Pineda, Maria Angeles Marques-Torrejon

**Affiliations:** ^1^ Department of Pharmacology, School of Pharmacy, University College London, London, United Kingdom; ^2^ Université Paris Cité, Inserm, CEA, Stabilité Génétique Cellules Souches et Radiations, LRP/iRCM, Fontenay-aux-Roses, France; ^3^ Université Paris-Saclay, Inserm, CEA, Stabilité Génétique Cellules Souches et Radiations, LRP/iRCM, Fontenay-aux-Roses, France; ^4^ Cell Signaling Lab, Department of Cell Biology and Histology, Faculty of Medicine and Nursery, University of the Basque Country (UPV/EHU), Bilbao, Spain; ^5^ Achucarro Basque Center for Neuroscience Fundazioa, Leioa, Spain; ^6^ Department of Medicine, Jaume I University of Castellon, Castello de la Plana, Spain

**Keywords:** tumor microenvironment, tumor cell invasion, residual tumor volume, radionecrosis, anti-angiogenic drugs, cytoreduction, glioblastoma spheroids, central nervous system

The central nervous system microenvironment is composed by different types of cells, including glia, immune and endothelial cells, and by soluble factors and components of the extracellular matrix (ECM). Together they safeguard a healthy functioning brain and support neuronal recovery upon injury or infections. However, during the evolution of a brain tumor, changes in the microenvironment can be coopted to sustain tumor growth. Indeed, the tumor microenvironment (TME) contains a mixture of cells including stem-like cells, reactive astrocytes, glioma-associated microglia and macrophages and myeloid cells, which generate a more favorable TME and increase tumor accommodation via secretion of soluble factors and formation of pro-tumoral cell-cell networks. Thus, the TME may contribute to tumor growth, invasiveness, stemness maintenance, therapy resistance and immune evasion, among others. For this reason, it is necessary to understand the pro-tumoral mechanisms modulated by TME to identify and develop new biomarkers and novel therapeutic targets to improve the overall management of neurological tumors.

The aim of the Research Topic “*Tumor Accommodation: The Importance of the Niche in Neurological Tumors*” was to generate a discussion regarding the role and the technologies to study the brain microenvironment in primary and metastatic tumors. Understanding the mechanisms and processes that cancer cells exhibit to adapt and survive in the brain is key to identify selective vulnerabilities. In this Research Topic we report the development and validation of an advanced *ex vivo* brain slice assay to model glioblastoma cell invasion into the complex brain microenvironment. Decotret et al. set up an *ex vivo* culture system in which human glioblastoma spheroids can be precisely implanted on murine brain slices. They embedded previously immunofluorescent stained brain slices into agar blocks and re-sectioned orthogonally to unravel the 3D axis and reconstruct intracerebral tumor invasion by confocal microscopy. This approach allowed the precise intracerebral characterization of tumor cell invasion, overcoming the limited resolution of single slice sections observed by traditional microscopy. Using this new methodology, the researchers visualized invasive structures beneath the tumorospheres that would otherwise go undetected and they were able to establish the presence of direct contacts of the cancer cells with alpha smooth muscle actin (α-SMA)-positive vasculature and glial fibrillar acidic protein (GFAP)-positive astrocytes in the microenvironment. Furthermore, they found striking differences in tumor cell motility when cancer cells were embedded in the *ex vivo* brain tissue versus, for example, when in contact with Matrigel. This result indicates that Matrigel might lack critical components that facilitate collective invasion and that the contact with the *ex vivo* brain tissue better mimics invasion observed in human tumors. Future work should focus on developing biofunctionalized hydrogels with different stiffnesses that can provide cancer cells with a defined microenvironment to better mimic invasion. Overall, these results highlight the importance of brain microenvironment and ECM in tumor invasion studies. Understanding motility and invasion of cancer cells and their interaction with the extracellular environment is key to tackle local tumor invasion in the surrounding tissue and also colonization of the brain from other parts of the body.

Two other works in this Research Topic reported clinical studies on patients with brain metastasis. Work by Baumgart et al. assessed the impact of residual tumor on overall survival of elderly patients with brain metastases. In this study, the authors determined the residual tumor volume by MRI at 72 hours post-surgical resection and surveilled the survival of patients. They found that, regardless of age or cancer type, residual tumor volume is a strong predictor for prolonged overall survival, with patients having had maximal cytoreduction surviving on average twice as long as the ones without complete resection. Brain metastasis secondary to other tumors are very common and can be treated with stereotactic radiosurgery. However, radiation necrosis is a serious complication associated with this procedure and strongly affects the brain microenvironment and the tumor niche. Work of Lolli et al. describes a case study of a 65-year-old female patient with bilateral brain radionecrosis six months after stereotactic radiotherapy. The patient suffered headaches and cognitive-motor impairments and later lung tumor progression, additional to the renal carcinoma she was initially treated for. For this reason, she was treated with the anti-angiogenic drug cabozantinib. Interestingly, two months after treatment, they found unpredicted effects on brain parenchyma, including volume reduction of the brain areas with radionecrosis and shrinkage of the associated edema determined by MRI. Thus, the case studies here reported, together with novel technologies, will help understand the role of TME in tumor progression and metastasis and will contribute to the identification of novel ways to target TME for therapeutic benefit. The list of tumor niche regulators is constantly growing, as it is the resolution and versatility of the techniques used to study their functions. We thus envisage that, in the future, modulators of the TME alone or in combination with current drug regimens may provide more effective therapies for brain tumors.

## Author contributions

RA: Writing – review & editing. LRG: Funding acquisition, Writing – review & editing. JP: Funding acquisition, Writing – original draft, Writing – review & editing. MM-T: Funding acquisition, Writing – original draft, Writing – review & editing.

